# An interesting case of crossed syndrome: ipsilateral facial paralysis with contralateral glossoplegia

**DOI:** 10.1186/s12883-023-03363-8

**Published:** 2023-10-17

**Authors:** Sangil Park, Su Hyeon Ha, Baeseoup Song, Ho Geol Woo, Sung Hyuk Heo, Dae-il Chang

**Affiliations:** Department of Neurology, Kyung Hee University Hospital, #23 Kyunghee-daero, Dongdaemun-gu, Seoul, 02447 Republic of Korea

**Keywords:** Crossed syndrome, Hypoglossal nerve, Glossoplegia

## Abstract

**Background:**

Stroke is rarely accompanied with peripheral facial paralysis and supranuclear palsy of the hypoglossal nerve. Both sides of the motor cortex innervate the hypoglossal nucleus; therefore, unilateral lesions of the upper motor neurons rarely result in contralateral lingual paresis. We report a rare case of crossed syndrome with associated hyperacute peripheral hemifacial paralysis and contralateral lingual paresis after a lower pontine tegmentum ischemic stroke.

**Case presentation:**

: A 73-year-old man presented with symptoms of hyperacute peripheral hemifacial paralysis. Upon protrusion, the patient’s tongue deviated to the contralateral side, without fasciculation or atrophy. Brain imaging showed focal ischemic stroke in the pontine tegmentum. However, lingual hemiparesis and multimodal neuroimaging findings differed.

**Conclusions:**

We suggest that cortico-hypoglossal fibers pass through the dorsal pontine. This case of crossed syndrome is a rare report of a lower pontine tegmentum ischemic stroke resembling an upper motor neuron lesion of the contralateral hypoglossal nerve.

## Background

Crossed syndrome is a form of hemiplegia with ipsilateral cranial nerve palsy and contralateral hemiplegia of the extremities, resulting from a unilateral brainstem lesion [1]. Lingual hemiparesis can result from damage to the hypoglossal nerve (lower motor neurons) and supranuclear innervation [upper motor neuron] [2]. Both sides of the motor cortex innervate the hypoglossal nucleus; therefore, unilateral lesions of upper motor neurons rarely result in contralateral lingual paresis [3]. Supranuclear lesions commonly result in central facial and lingual paralysis, while crossed paralysis is a relatively uncommon symptom. Herein, we report a rare case of crossed syndrome with associated hyperacute peripheral hemifacial paralysis and contralateral lingual paresis after lower pontine tegmentum ischemic stroke.

## Case Presentation

**Fig. 1:**
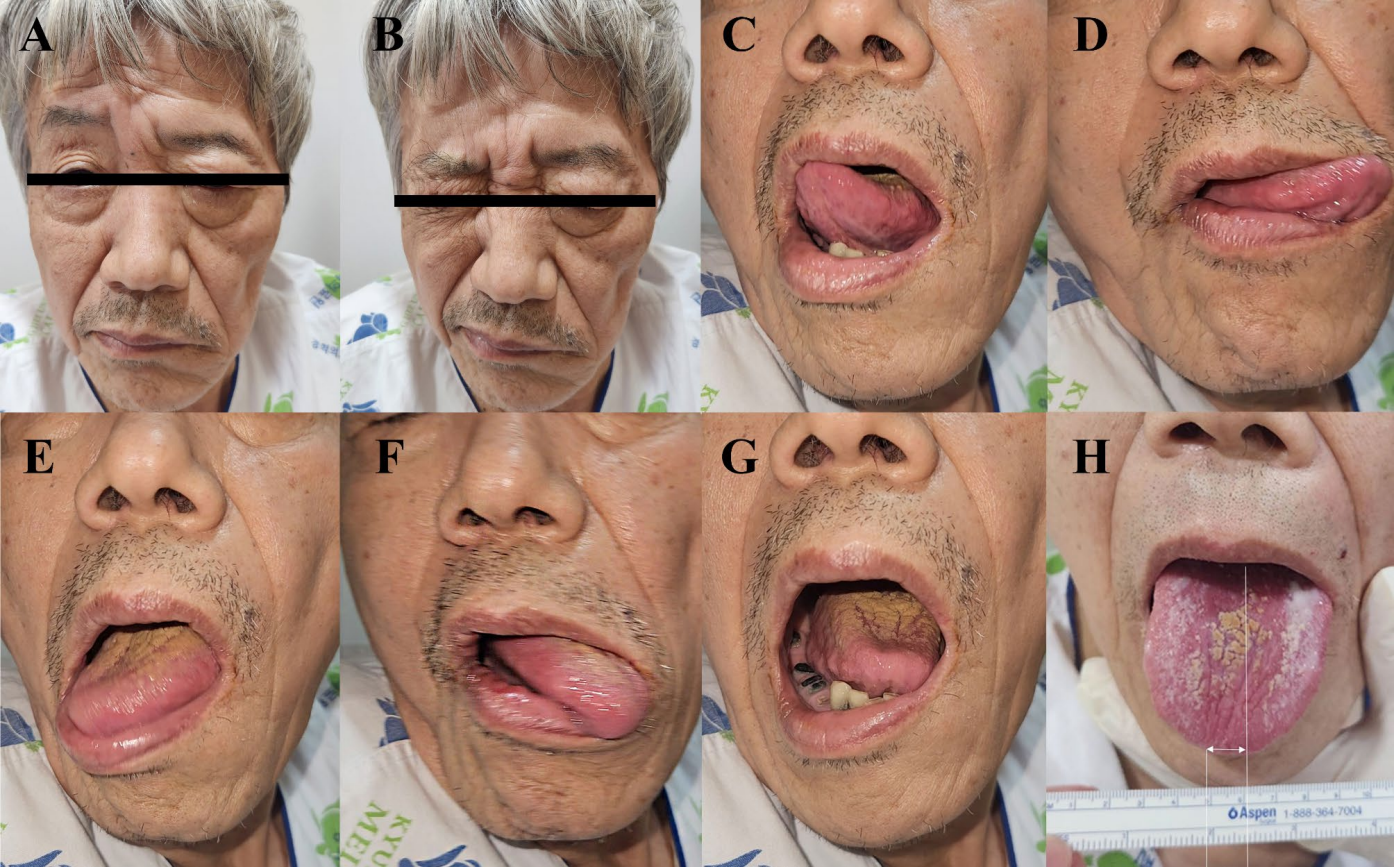
Left-sided peripheral facial nerve palsy and evidence of an upper motor neuron lesion. There is reduced innervation of the forehead (**A**), and impaired lid closure when the eye is closed (**B**). The tongue had a normal range of motion during movement in different directions (**C**-**F**) and at rest (**G**). On tongue protrusion, there is a right lingual paresis (**H**)

A 73-year-old man presented with a sudden onset of left facial paralysis. Patient visited the emergency department 5 h after the onset. The patient was on medication for hypertension, which was diagnosed several years prior; and the patient was a heavy smoker. The patient had no apparent history of head or neck trauma. Neurological examination revealed profound left peripheral facial paralysis **(**Fig. 1A, B**)**. Upon protrusion, the tongue deviated to the right, without fasciculation or atrophy. However, the tongue’s range of motion was normal at rest and did not protrude in any direction **(**Fig. 1C-H**)**. It was suspected that there was a lesion in the upper motor neuron of the left hypoglossal nerve. An intraoral examination revealed no abnormalities in the uvula or palate. No complaints of swallowing difficulty or any motor or sensory impairments of the extremities. Although the patient’s blood pressure was high (169/89 mmHg), other vital signs were stable.

**Fig. 2:**
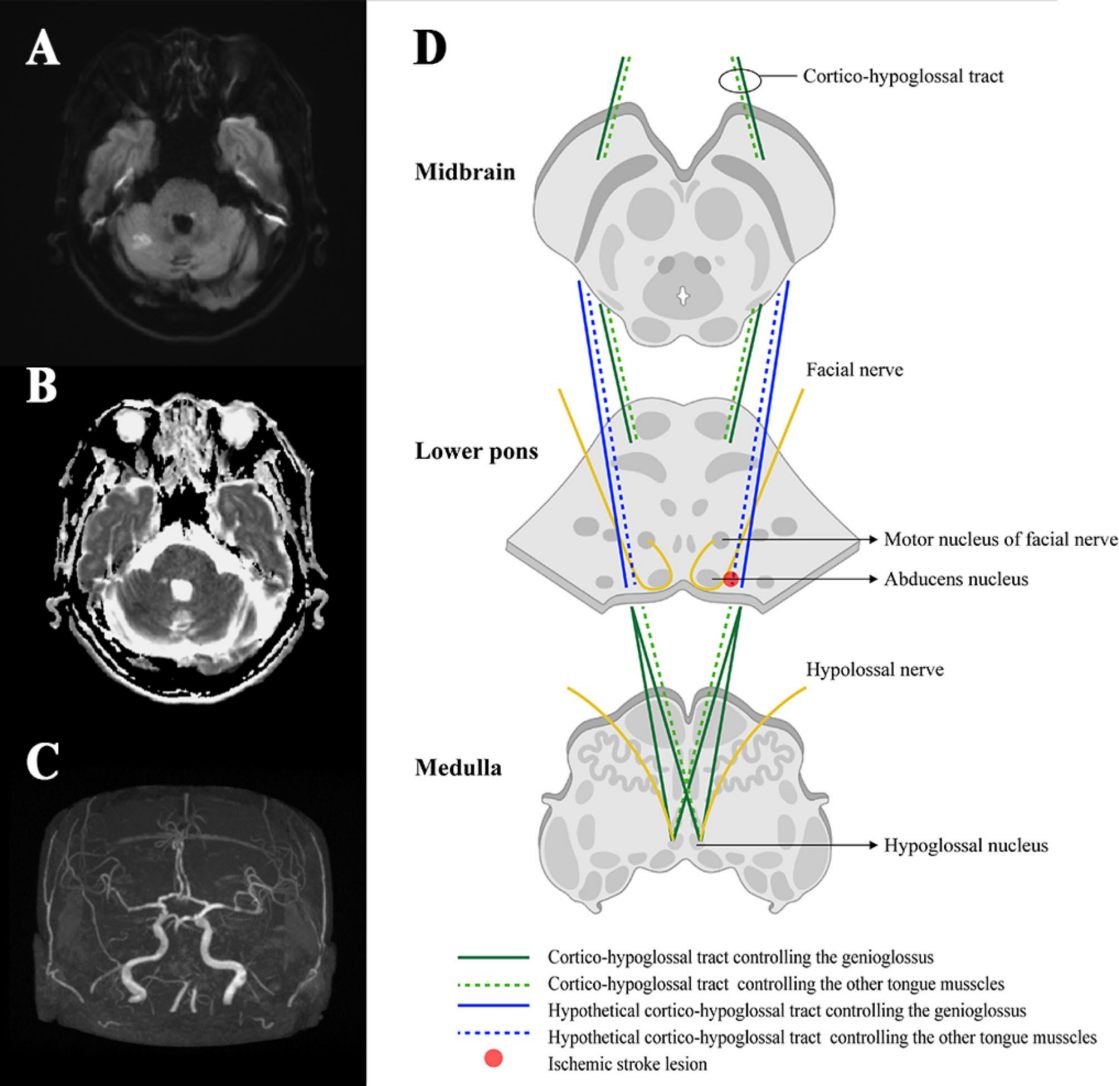
Focal acute ischemic lesion in left pontine tegmentum, axial diffusion weighted images (**A**) and apparent diffusion coefficient (**B**). There is a basilar artery occlusion on magnetic resonance imaging (**C**). The hypoglossal nucleus receives corticobulbar fibers from both hemispheres for voluntary control (**D**). We suggest a unique hypothetical cortico-hypoglossal tract (**D**)

Acute left caudal pontine stroke was suspected, and a neuroimaging diagnostic workup was performed immediately. Initial brain computed tomography revealed no ischemia or hemorrhagic lesions. However, brain magnetic resonance imaging revealed a focal hyperintense lesion in the left pontine tegmentum on diffusion-weighted imaging **(**Fig. 2A**)**. A low signal intensity on the apparent diffusion coefficient map demonstrated that the lesion was an acute ischemic infarction **(**Fig. 2B**)**. Therefore, we determined that the lesion affected the upper motor neuron of the right hypoglossal nerve and the lower motor neuron of the left facial nerve.

Occlusions were observed in the fourth segment of the vertebral and basilar arteries, as identified by magnetic resonance angiography **(**Fig. 2C**)**. Although the occlusions were initially attributed to large artery atherosclerosis, considering other etiologies, including cardioembolic causes, is important. Transthoracic echocardiography, 24-hour Holter monitoring, and transcranial Doppler ultrasonography-bubble test were performed, but no other embolic causes were identified. The lesion was relatively small in size compared to the area of occlusion and the patient experienced only mild symptoms; therefore, dual antiplatelet therapy rather than thrombectomy was recommended to prevent a secondary stroke. The patient received dual antiplatelet therapy (aspirin 100 mg/day and clopidogrel 75 mg/day) and high-intensity statin therapy (atorvastatin 40 mg/day). On the seventh day of hospitalization, the tongue deviation noticeably improved, and the patient was discharged without any worsening neurological symptoms.

## Discussion and conclusions

The hypoglossal nucleus is located on the medial medulla, immediately below the fourth ventricle **(**Fig. 2D**)**. It receives corticobulbar fibers from both hemispheres for voluntary control and is responsible for reflex movements such as chewing, sucking, and swallowing [2]. However, supranuclear palsy of the hypoglossal nerve can result in motor impairment on the contralateral side of the lesion, even without atrophy or fasciculations. This is because among the extrinsic muscles of the tongue, only the genioglossus (which is responsible for tongue protrusion) crosses the unilateral innervation [4]. Therefore, if the tongue deviates only during protrusion, without atrophy or fasciculations, a lesion in the upper motor neuron on the contralateral side is likely. However, the absence of atrophy or fasciculation does not exclude the involvement of lower motor neurons. Regarding the timing of the injury, a neurological examination is necessary because it may be too early to observe atrophy or fasciculation.

Corticobulbar fibers descend from the peri-sylvian area of a motor homunculus through the corona radiata, internal capsules, and cerebral peduncle to the basis pontis, and the opposite projections cross the midline at the pontomedullary junction [5]. However, dorsal pontine lesions do not involve cortico-hypoglossal projections [6].

In previous studies, the decussation of the cortico-hypoglossal tract was generally located at the pontomedullary junction, and variations were due to individual differences [7, 8]. Furthermore, before decussation, cortico-hypoglossal fibers pass through the medial portion of the ventral pons and the lateral portion of the pons without crossing the midline [6]. To the best of our knowledge, this is a rare report of crossed syndrome with ipsilateral facial paralysis and contralateral glossoplegia associated with a lower pontine tegmentum ischemic stroke resembling a supranuclear lesion of the hypoglossal nucleus.

We suggest that cortico-hypoglossal fibers pass through the dorsal pontine, although this occurrence is exceedingly uncommon **(**Fig. 2D**)**. It is crucial to acknowledge that the presentation of symptoms can vary, contingent upon the precise location and extent of the lesion within the supranuclear pathway. Consequently, studying similar cases is of paramount importance to enhance our comprehension of the wide-ranging patterns of cortico-hypoglossal fiber connections and their correlation with clinical manifestations. Therefore, additional investigations involving more cases are necessary to shed light on the underlying mechanism responsible for the rare symptoms observed in our patient.

If the tongue deviates to one side during protrusion, without associated atrophy or fasciculation, clinicians should consider contralateral lesions of the upper motor neurons and note that there may be various patterns of cortico-hypoglossal fibers. Furthermore, the possibility of acute stroke should not be excluded in older patients, patients with risk factors for vascular disease, and patients who have sudden symptoms; and brain imaging studies are necessary.

## Data Availability

The datasets supporting the conclusions of this article are included in the article.
